# Biosignal Sensors and Deep Learning-Based Speech Recognition: A Review

**DOI:** 10.3390/s21041399

**Published:** 2021-02-17

**Authors:** Wookey Lee, Jessica Jiwon Seong, Busra Ozlu, Bong Sup Shim, Azizbek Marakhimov, Suan Lee

**Affiliations:** 1 Biomedical Science and Engineering & Dept. of Industrial Security Governance & IE, Inha University, 100 Inharo, Incheon 22212, Korea; trinity@inha.ac.kr; 2 Department of Industrial Security Governance, Inha University, 100 Inharo, Incheon 22212, Korea; 22192221@inha.edu; 3 Biomedical Science and Engineering & Department of Chemical Engineering, Inha University, 100 Inharo, Incheon 22212, Korea; busraozlu17@gmail.com (B.O.); bshim@inha.ac.kr (B.S.S.); 4 Frontier College, Inha University, 100 Inharo, Incheon 22212, Korea; 219730@inha.ac.kr; 5 School of Computer Science, Semyung University, Jecheon 27136, Korea

**Keywords:** mouth interface, voice production, artificial larynx, EMG, biosignal, deep learning

## Abstract

Voice is one of the essential mechanisms for communicating and expressing one’s intentions as a human being. There are several causes of voice inability, including disease, accident, vocal abuse, medical surgery, ageing, and environmental pollution, and the risk of voice loss continues to increase. Novel approaches should have been developed for speech recognition and production because that would seriously undermine the quality of life and sometimes leads to isolation from society. In this review, we survey mouth interface technologies which are mouth-mounted devices for speech recognition, production, and volitional control, and the corresponding research to develop artificial mouth technologies based on various sensors, including electromyography (EMG), electroencephalography (EEG), electropalatography (EPG), electromagnetic articulography (EMA), permanent magnet articulography (PMA), gyros, images and 3-axial magnetic sensors, especially with deep learning techniques. We especially research various deep learning technologies related to voice recognition, including visual speech recognition, silent speech interface, and analyze its flow, and systematize them into a taxonomy. Finally, we discuss methods to solve the communication problems of people with disabilities in speaking and future research with respect to deep learning components.

## 1. Introduction

Voice is a basic means of communication and social interaction through spoken language. People with voice disorders face serious problems in their daily lives, which may lead to emotional instability and isolation from society. A voice disorder implies that the pitch, intensity, or fluidity of one’s voice does not conform to his or her gender, age, body composition, social environment, and geographic location. In other words, the term voice disorder refers to all abnormal conditions in which the expression of the voice is not in its normal range. In the human body, voice is produced by the vibration of the vocal cords as a result of the airflow supplied by the respiratory system, thus the normal voice production depends on the coordination among airflow, laryngeal muscle strength and the supraglottic resonator cavities such as pharyngeal, oral and nasal cavity [1]. The reasons for the voice disorder can be categorized as organic, functional, and/or psychogenic causes. Organic causes may have structural (vocal fold abnormalities, inflammation or trauma to the larynx) and neurologic (recurrent laryngeal nerve paralysis, adductor/abductor spasmodic dysphonia, Parkinson’s disease, multiple sclerosis) origins, while functional causes may arise from phonotrauma, muscle tension dysphonia, ventricular phonation, and vocal fatigue. Anxiety and depression are considered psychogenic causes of voice disorder [1]. Although there are several approaches for the treatment of voice disorder, such as PMA using articulatory data captured from the lips and tongue [2] or a contactless silent speech recognition system using an impulse radio ultra-wideband (IR-UWB) radar [3], the communication options are limited for the patients whose larynx (voice box) is surgically removed following throat cancer or trauma [4]. To overcome this problem, the external device, called electrolarynx, has been developed as a form of artificial larynx communication where an electronic vibration is produced and resulted in a monotonic sound that will be formed to speech [4]. However, the unpractical use and unnatural voice production make the device challenging for the patients. Thus, a novel concept must be developed for voice recognition and production technologies which also can include brain-computer interfaces (BCIs) and silent-speech interfaces (SSIs). SSI is considered as a plausible approach to producing natural-sounding speech by capturing biosignals from the articulators, neural pathways, or the brain itself in brain-computer interfaces (BCIs) [5,6,7,8]. Recently, various biosignals captured by techniques such as ultrasound, optical imagery, EPG, EEG, and surface electromyography (sEMG) have been investigated in terms of developing silent speech communication systems [8,9,10]. 

**Figure 1:**
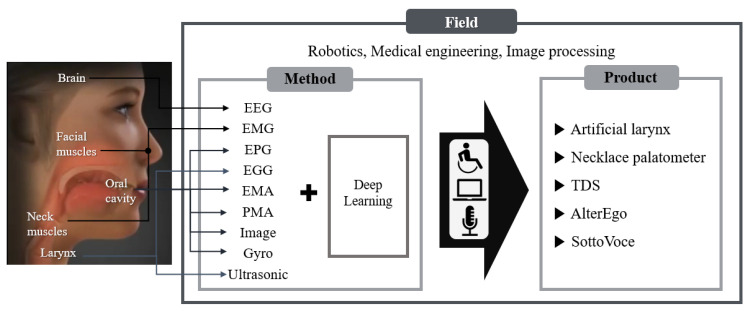
How to help people with voice disorders communicate with others: to recognize voices, which indicates that biosignals are processed via various methods and transmitted to devices suitable for the user, and can be used in several fields, such as robotics, medical engineering, and image processing.

In this review, as summarized in Figure 1, we report on the recent advances in the field of biosignal-based voice recognition and production with deep learning-based technologies. We first introduce sensor technologies that can acquire voice-related biosignal data as well as their applications in voice recognition and production. Regarding deep learning, we also present not only voice recognition technology through sound or image but also silent speech representation interface. We concluded with a summary and perspectives on the current and future applications.

## 2. Biosignal-Based Speech Recognition 

Biosignals provide the information belong to the electrical, chemical, mechanical, and biological processes of the living organism [11,12]. In the field of biosignal-based speech recognition, signals from brain, muscle and movement are considered as potentially useful biosignals that can be measured by different techniques. Traditional acoustic sensors capture the sound pressure waves resulting in acoustic biosignals. In case of the absence of acoustic output, other speech-related biosignals can be acquired and processed, called silent-speech recognition. Some of the methods used to acquire speech-related biosignals that occur during speech production are given in Table 1. These signals can be used in automatic speech recognition (ASR) systems which convert speech into text. In conventional ASR systems, acoustic input is received through a microphone and analyzed by some model or algorithm, followed by the generation of output in the form of a text [12,13]. These technologies can be found in a large variety of applications from virtual assistants installed in mobile devices to hands-free computing [14]. However, there are some drawbacks related with the ASR systems, such as the rapid degradation of performance in the presence of ambient noise, lack of privacy, and limited usage for those with speech disorders [8,10]. To overcome some of these limitations, silent-speech recognition stands as an attractive approach, which depends on the acquisition of speech-related biosignals, also referred to as biosignal-based speech recognition [10,11].

In this section, methods used to acquire speech-related biosignals and their usage in speech recognition will be presented, with the specific emphasis on signals from muscle, brain, and articulatory activities.

### 2.1. Muscle Activity

#### EMG-Based Speech Recognition

**Figure 2:**
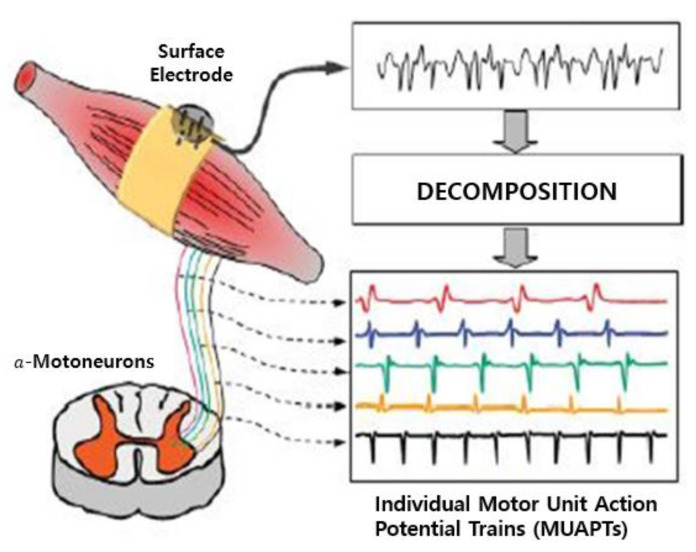
Measurement of muscle electrical signals using EMG technology.

EMG is a technique in which the electrical activity produced by muscles is recorded using electrodes either attached to the skin or inserted into the muscle (Figure 2). Since the usage of surface electrodes offers a non-invasive way of recording, sEMG is generally preferred over the EMG with needle electrodes. sEMG evaluates the muscle’s function by observing the electrical activity that emanates from speech musculature around the region of face and neck. These signals can be used in ASR systems and overcome some limitations by being able to recognize speech in very loud environment [19], to be speech interfaces for those with speech disabilities [11] and to improve performance by combining acoustic signal [20,36].

**Figure 3:**
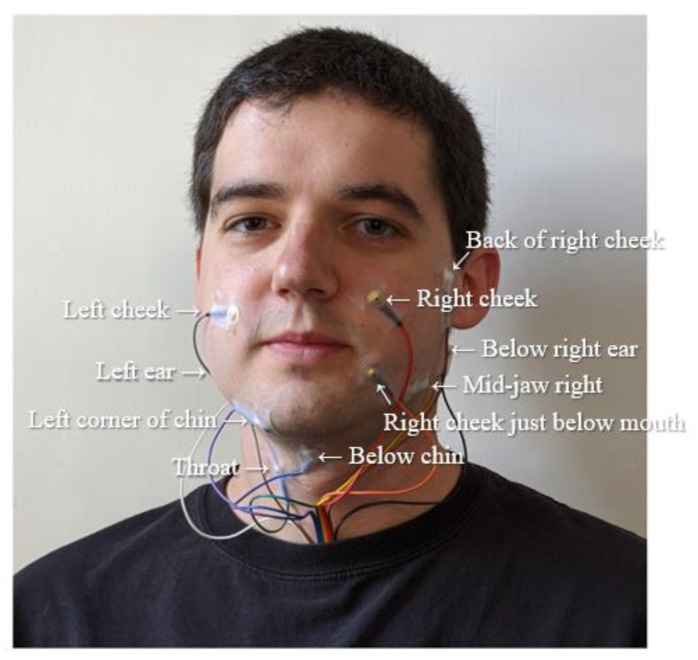
Examples of the positions for electrodes on a face [25].

Early studies of the EMG-based speech recognition goes back to 1985, with the system developed for revealing five Japanese vowels in real-time [21]. In this early study, the accuracy of word recognition was only 60%. However, more research has been carried out onwards. In 2011, Schultz [22] recognized plosive and fricative noises in the input voice. Then, she proposed a coarticulation modelling for the word recognition by selecting a trained classification based on those noises. They, in turn, proposed modelling to reduce the natural language error rate of 101 words by up to 10%. In 2003, a word recognition using the shape of the mouth for vocal dysfunction has been continuously studied [37], but it has not been successful [23]. In 2010, Janke carried out the word recognition by using the spectral mapping method but it is failed recognizing words with only 53% of accuracy. Then, in 2014, Srisuwan [24] used the moving average filter and stacking filter as the word recognition method improved the accuracy of 11 words by 78%. However, there is an extra for use in a smaller number of words. Currently, research on word recognition using EMG signals for various languages such as Thai, Chinese, and English is progressing [15,24,38]. Figure 3 shows the positions for electrodes on a face [25]. 

**Figure 4:**
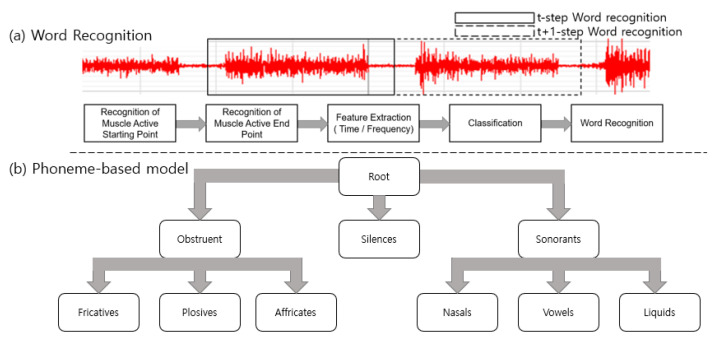
Example of speech recognition using the EMG signal.

More recently, a study on real-time speech conversion system using EMG signal was also conducted [16]. In addition, there are studies that have improved the accessibility of data collection by using a patch electrode different from the electrode used in the past [17]. Pattern recognition and machine learning tools are used in signal processing for word recognition. EMG signals are obtained from the facial and neck muscles. In order to recognize isolated words and the tones of the word, speech-related features in time domain and frequency domain are extracted, and after that, the classifier is used to recognize words and tones [24]. In the process, the algorithms were developed by evolving speech recognition models: for recognizing sequences of words using grammar models, and finally for recognizing a vocabulary of previously untrained words using phoneme-based models [10]. Figure 4 shows an example of word recognition using the EMG signal. 

### 2.2. Brain Activity

#### EEG-Based Speech Recognition

EEG is a technique to record the electrical activity of the brain. EEG signal is carried out when an instruction is transmitted to or received from emotional, visual, auditory, sensory, or muscular stimuli. Therefore, the research in the EEG signal is carried out in various fields such as robotics, medical engineering, and image processing [39,40,41]. EEG signal is used to recognize the voice by measuring its frequency through potential differences generated from the temporal lobe [8,9]. The temporal lobe responds when the auditory stimulus due to the presence of the acoustic area and auditory cortex. Hence, the audible frequency changes are measured through the EEG signal when the voice is generated, and the frequency range of the currently heard voice is recognized. The limitation, however, has been revealed that the noise and the true signal may not be separated for EEG since when humans go astray mentally even when he/she is in the middle of his/her own speechless.

In the human computing interface (HCI) field emotion recognition is becoming a core technology, and voice and image data are used as the external emotion expressions. However, since there is a disadvantage that a person can intentionally create false data, the research using EEG signal data can be limitedly adopted for human emotion recognition. Recently the attention has been focused on a study using a change in tone or accent of a voice in combination with voice data. Therefore, the emotion classification based voice tone research using EEG signal and voice data is introduced for natural voice reproduction [27,28].

In addition, research about the human voice recognition [42] and gender recognition [43] is being conducted but not yet to the level of the full sentence recognition with emotions. The detailed action potential for cranial nerves can’t be recorded because the EEG signal is measured mainly through the scalp. Although it is possible to recognize the large range of frequency, it is still difficult to recognize the detailed range of the words, mouth movements, vocal movements.

### 2.3. Articulatory Activity

#### 2.3.1. Correcting Motion Interface

**Figure 5:**
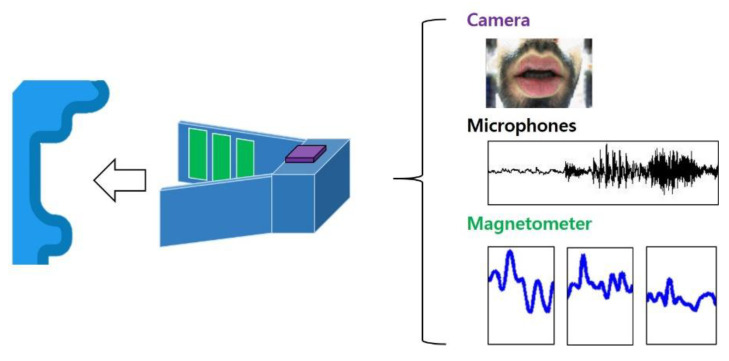
Collect the subject’s tongue movements, lips movement, and voice data with the development device for the wireless tongue tracking technique combining the camera and several acceleration sensors [35].

Articulation disorders (AD) is a speech disorder that affects the movement of the tongue, lips, and jaw. This may be caused by less developed tongue exercise of the interlocking disorder between the upper and lower lips, or if the pronunciation is not clear due to abnormal lower jaw, etc. As a result, communication problems arise, and therapies for this disorder are repeated through the patient’s pronunciation and movement correction. Of these, the tongue plays the largest part in the conversation. Because the pronunciation is mainly influenced by the tongue, it is the place where the correction treatment has most frequently been. However, the movement of the tongue is obscured, so it is not easy to correct. Therefore, studies are being conducted to observe the movement of the tongue according to the user, to acquire data, and to train the correct tongue movement and speech method [35,44,45]. Figure 5 represents the subject’s tongue movements, lips movement, and voice data with the development device for the wireless tongue tracking technique combining the camera and several acceleration sensors.

The gyro sensor basically measures the angular velocity of an object and is currently built-in satellite, drone, virtual reality (VR), wearable device, smart device, and so on. When the angular velocity obtained from the rotating object is integrated by the shifted time, it is possible to measure the inclined angle [44]. Applying this advantage to the human tongue, the angular velocity x, y, z value, and angle change can be detected according to the pronunciation. However, since the gyro sensor is susceptible to temperature, the errors may occur in integrating the sensor values, and the sensor drift phenomenon may also occur. Therefore, the gyro sensor may be used together with an acceleration sensor so that the errors can be minimized.

The 3-axial magnetic sensor is a sensor that measures the acceleration of an object. It can measure not only acceleration but also various physical properties such as shock, vibration, and trembling. As with gyro sensors, it is used in a variety of transportation systems, factories, robots, and smart devices [45]. Acceleration values are integrated to obtain information on the velocity and the movements of the object. If the acceleration is integrated with respect to time, the value of the displacement can be calculated. By using these physical characteristics and attaching the acceleration sensor to the tongue, it is possible to detect numerical values such as vibrations, trembling, and speed when pronouncing voices. At present, wireless tongue tracking technology combining camera modules like several acceleration sensors has been developed and studies are being carried out to identify the user’s phonemic markers [35].

#### 2.3.2. EPG-Based Speech Recognition

EPG is the technology which visualizes the location and timing of tongue contact with the pseudopalate inside the mouth [29,30] and used as an effective tool to diagnose and treat a variety of speech disorders [46]. EPG technology has been developed over about 50 years, and initially, it started to process the signals according to the reaction with symmetrically arranged pairs of electrodes. Then, by adding a model for the electrode channel and voice information, more sensitive and detailed stimulus signals are received and converted into voice signals, enabling more precise voice output. An artificial palate containing an electrode is transplanted into a patient. When being touched for a part of the artificial palate, an electrical signal at the contact area is sensed, so that the portion of the tongue can be detected according to the pronounced and the changed shape in the patient mouth [45].

Russel et al. use the contact information between the tongue and the palate with the help of pattern recognition and feature selection techniques. In this study, they used a custom-made pseudopalate with 118 touch sensors and tested the 50 different English words by different recognition techniques, bringing the recognition rates up to 78% [47].

In recent years, a mini and convenient device such as a necklace palatometer included a microphone and a portable training unit (PTU) have been generated, and the potential to develop into a commercial medical device in the future is almost feasible [23]. In addition, some technologies are required such as biosignal measurement content, fast signal stimulation, miniaturization, and construct a convenient interface for the user. Electrodes are implanted not only on the palate and tongue but also on the lips and jaw [18]. The current developed technologies, however, have a limitation in detecting the precise stimulation of the response between the palate and the tongue. Specifically, there are sounds that the tongue and the palate would not touch like ‘r’ sound which needs to be dexterously detected. If it produces a voice that is consistent with the movement of the corresponding pronounced muscle movement, it can be a great effect for a more accurate voice implementation which is up to the eagerness of the dysfunction vocal patients [48].

#### 2.3.3. Magnetic Articulography-Based Speech Recognition

##### Electromagnetic Articulography (EMA)-Based Speech Recognition

EMA is used to detailly investigate speech articulation. In EMA, magnetic field sensors are placed on main articulators and a magnetic field is produced by coils that are positioned around a participant‘s head [49]. This system can be used to determine the complete vocal tract configuration with precise tracking of the sensors’ locations in a 2D or 3D Cartesian space [11]. However, EMA is an invasive procedure and not suitable for use in everyday life because of the wires that run inside the mouth.

Heracleous et al. [50] developed ASR based on articulation information by using EMA. Movements of the articulators are tracked by EMA and are used in hidden Markov models (HMMs). The vowel, consonant, and phoneme accuracies were found to be 93.1%, 75.2%, and 78.7%, respectively, by using only EMA parameters without any audio information. Additionally, experiments were performed in a simulated noisy environment using EMA parameters and fused noisy audio data. Two decision methods were selected as multi-stream HMM decision fusion and late fusion. For a feature fusion method, concatenative feature fusion was selected. They reported that the integration of EMA parameters significantly increased recognition accuracy.

##### Permanent-Magnetic Articulography (PMA)-Based Speech Recognition

PMA is a technique for recording the articulatory movements based on sensing the changes in magnetic field. There are two main properties that make PMA different than EMA. First, the location of emitters and sensors are reversed in PMA. Small permanent magnets are placed on speech articulators and the generated magnetic field is captured by sensors arranged on a wearable frame. Secondly, there is no need for wires leading out of the speaker‘s mouth which makes PMA more comfortable than EMA. 

Hofe et al. [49] reported a silent speech interface for small vocabulary recognition based on PMA. They demonstrated the potential usage of the system to capture phonetic detail for speech recognition. Word accuracies were found to be above 90%. Since the system was designed for simple tasks, it was suggested to create a hybrid system between PMA and other techniques such as EMG, BMI, and optical lip reading for an effective silent speech system.

### 2.4. Application of Device Interface

#### 2.4.1. Interface Technologies by Tongue and Mouth Sensors

**Figure 6:**
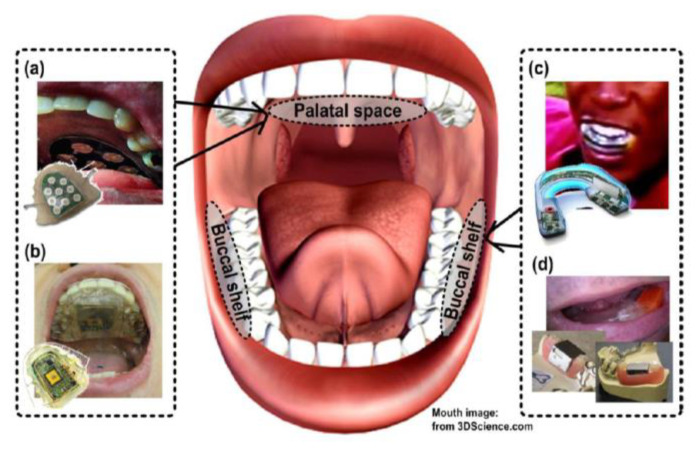
The interface is attached to the palate (iTDS-1) [33].

A device called tongue drive system (TDS) can be used for communicative expressions has been developed. A tongue is chosen as a tool to help the voice restoration since it is more suited for control. Besides, the tongue is composed of strong and dexterous muscle fibers that will not easily become fatigued. Even if it is a starting phase, the TDS is a promising approach for the patients with spinal cord injury that will still be able to function their tongue, because it is connected to the brain through cranial nerves directly. In the future, these functions will be implemented in the form of SoC (System on Chip) which can be small-sized and placed inside the mouth. Figure 6 illustrates a device with the function of communicative expression which uses the tongue to press the keypad attached to the palatal space [33]. 

By combining these technologies with EPGs, more accurate, active, self-evolving technologies can be developed. Electrodes implanted into the palate in the EPG as shown in circular dots. When the tongue and palate touch, the more sensitive, the darker the color [31]. Because the tongue and the palate contact of everyone is a little differently even if the person pronounces the same pronunciation [32]. Through frequent experiments, the pattern by the person is transmitted to the device through the interface to generate a unique accurate voice [26] and can realize self-voice expression.

#### 2.4.2. Internal Implantable Devices

Basically, the biosignal based interface technology uses an artificial biosignal such as an electromyogram and an electroencephalogram. It refers to a technology that is used by a patient as a HCI using a computer in vitro or a driving control device implanted in the body [51]. The sensor is attaching to the body and mainly used as an interface for the disabled. The transplanted device is an EMG that senses the signal by the muscle movement. Through a series of processes (amplification, signal conversion, integration, etc.) the signal detection through the nerve stimulation with the signal detection transmits to the electronic devices such as a computer in vitro or in vivo to provide signal analysis and result processing to the user.

By measuring the inductance change of the coil as the core of the ferromagnetic coil attached to the tongue moves, EMG detects the movements of the tongue using electrical switching, the Hall-effect detection, and the pressure sensing. The system consists of a ferromagnetic material attached to the tip of the tongue and an induction coil attached to the side of the mouth. The user gives control of command by moving the tongue of its specific positions, such as touching the part of the tooth in the tip of the tongue. The tongue can be moved definitely and quickly in the mouth, for the disabled who use the wheelchair can easy to control and have relatively accurate movements. The application of such an interface device related to a voice like EMG or EPG can be expected to improve the accuracy and the long-term usages due to implantation in the body.

A self-tracker [34], which implanted in the tongue, wireless receiver, 3-axial magnetic sensor module and the eTDS consisted of a control unit, represents a case of the permanent magnet made into the size of a mucous membrane in the tongue. As the tongue moves, a magnetic field is generated by various magnetic traces around and inside the tongue. These changes are detected by magnetic sensors installed in the headset. The sensor output is wirelessly transmitted to a smartphone and a personal digital assistant (PDA). A signal processing algorithm operating on a PDA for sensor signals and conversions by a user control command, and the signals are wirelessly transmitted to devices (wheelchair, computer, TV) suitable for the user’s environment. The advantage of TDS is that it has a small number of sensors and a small magnetic tracker that can capture or express special commands by closely capturing the movement of the tongue.

As for the communicative expression technology using the movement of the tongue is developed in the Georgia Tech Bionics lab (GT-Bionics, Atlanta, GA, USA) by Ghovanloo [52]. The device is wireless and is designed to be not overtaking off and made in a wearable format. In this research, the wheelchair and computer operation is possibly made through this technology, but voice recognition has not been implemented in this TDS.

#### 2.4.3. Language Interfaces

**Figure 7:**
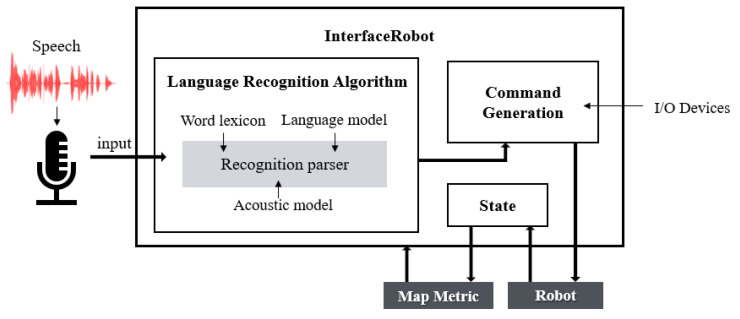
Voice remote control system structure based on language recognition processing.

In a language-based interface, as shown in Figure 7, the user uses a common microphone to record voice commands and the signals are controlled and analyzed through a language recognition algorithm [53]. Generally, language recognition requires the training that consists of the recording of voice commands and their subsequent systematic classification.

According to the thesis [54], a relatively good control interface can recognize 15 voice commands and reach a fairly high success rate of 96%. A major flaw in the language-based interface is that it is very sensitive to ambient noise, making it difficult to differentiate and classify signal to noise. To address this, a microphone voice activity detection (VAD) scheme [55] that enhances performance in a variety of noise environments in consideration of the sparsity of the speech signal in the time-frequency domain is proposed. And the language-based interface with the robot’s control is developed [56] and it reduced the ambient noise by 30%, the resulting inaccuracy has been improved.

## 3. Deep Learning Based Voice Recognition

Voice recognition refers to the process of identifying words or word columns and extracting meanings by entering voice waveforms. The commonly used voice recognition algorithm showed better performance than conventional acoustic models by using a deep neural network (DNN) [57] or combining it with the previously used hidden Markov model (HMM) [58]. Also, the studies are actively conducted to improve the accuracy of voice recognition results using an end-to-end learning method [59,60,61,62,63,64,65] that output a series of characters or words by inputting a series of audio features without any unit conversion or using a recurrent neural network (RNN) [60,61] that can well reflect the grammatical structure. Because RNN is mainly used for sequence data, and convolutional neural networks (CNNa) are mainly used for processing data with images or videos [62,65], wherein each case different networks are used for good voice recognition results [64,66,67,68]. Elements that constrain speech recognition performance vary widely from speaker characteristics such as pitch, accent or speech rate to interfering signals that make it difficult to distinguish the actual voice such as the background noise, channel distortion, or acoustic echo. Among them, in relatively little noise environments, robust automatic speech recognition works well but there are problems when reverberation or other complications exist [69], so eliminating background noise is important in improving speech recognition performance from audio communication channels [70]. Well-known noise reduction techniques are spectral subtraction [71] method based on the direct estimation of the short-term spectral magnitude, spectral masking [72] using weights the frequency sub bands of the mixed-signal for separating speakers and statistical methods [73] based on Wiener filtering [74]. Also, because voice recognition performance is sensitive to the size or the quality [75] of the voice database, studies are being conducted to improve learning speed and performance. 

If the auditory signals cannot be used as input when recognizing the voice, the natural voice should be synthesized through the movement of the mobile organs of the vocal tract (e.g., tongue, lips). The system that performs automatic articulatory-to-acoustic mapping can be a component of these silent speech interfaces (SSIs) [6], and the studies are underway to solve the problems of not being able to record the voice signal itself [76] and reconstructing the voice by converting the articulatory movement of the voiceless patient into speech [77]. Table 2 presents a brief summary of the deep learning models so far.

### 3.1. Visual Speech Recognition

The range of audible frequencies a person can listen to is 20 to 20,000 Hz, and a person can create and understand whispered or murmured speech at very low signal levels. Also, many people can understand a few words using only visual information such as lip-reading. Generally, there are methods of inferring speech using the lips’ visual information, either an image analysis-based method of detecting the movement of the lips or a method of guessing what the speaker is saying as the speaker’s mouth changes. However, these methods are usually sensitive to lighting variations [92] and the traditional approach to lip-reading has only limited vocabulary because simply reading the lips alone cannot predict all sounds spoken by the speaker (e.g., those with very similar lip movements). But more recently, visual speech recognition research, which aims to recognize images and analyze speech without audio, to overcome the limitations of this lip-reading using deep learning, such as RNN, CNN, and end-to-end DNN, are increasing. In other words, an initial system dealing only with simple recognition tasks, such as alphabet or digit recognition, led to a system that gradually implemented more complex and continuous lip-reading, such as recognizing words or sentences [93]. The goal is not just to read the changing region of oral expressions, but to solve problems with databases getting more and more and complex caused by increasing the number of speakers, diversity of posture, and the lighting variations [94], and to increase awareness to obtain information related to speech messages by taking into account the facial expressions of a person or by deciphering spoken information using the context of a conversation [95].

#### 3.1.1. LipNet

**Figure 8:**
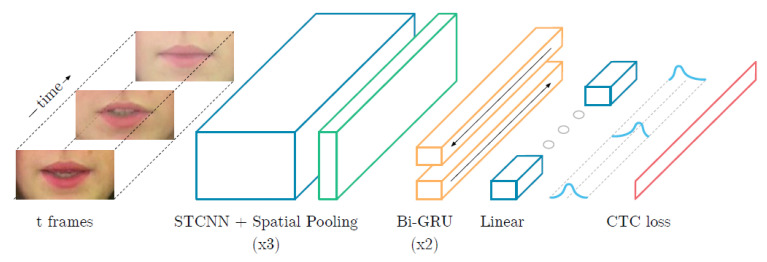
LipNet architecture [64].

LipNet [64] is a lip-reading system developed by DeepMind Team, a Google artificial intelligence development company (London, UK), and researchers at Oxford University in the UK. LipNet is an end-to-end sentence-level lip-reading model, which predicts the full-sentence sequence of what lips of the person pronouncing the sentence when they see the shapes of the movements and informs the results in text. The architecture of the LipNet is shown in Figure 8, it is characterized as the first model designed to simultaneously learn spatial-visual features and sequence models. When the features are extracted through the spatiotemporal convolutional neural networks (STCNN) with the sequence of the video frame initially as input, the results are processed using a bidirectional gated recurrent unit (Bi-GRU) for efficient temporal aggregation. Then, after applying a linear transformation for each output, connectionist temporal classification (CTC) is used to train without alignment information between the input data and the label, as a result, the variable-length sequence of video frames is converted to text sequences. In this case, only phonologically important parts of mouth form have been emphasized and qualified using the saliency visualization technology. LipNet, consisting of such neural network architecture, has been tested to demonstrate that it is more recognizable than the lip-reading skill of deaf people when translating the same sentence, but there is a limit to measuring accuracy with specially designed videos with well-trained BBC news cases for testing.

#### 3.1.2. Automatic Lip-Reading System

The model presented in [90] for lip-reading is the hybrid neural network architecture, which combines CNN with attention-based long short-term memory (LSTM) to implement automatic lip-reading. When a moving video is received as an input, it is separated from the audio signal and extracted about 25 key-frame per second from the sample video and going through the oral image segmentation phase of finding the mouth area using the key points of the mouth. The data is then processed in pixels, where spatial characteristics are extracted using a VGG network of CNN to overcome image deformation. In order to pick out valid information, sequential information and attention weights are studied through the attention-based LSTM, and then predict the final recognition results by the fully-connected layer and the final SoftMax layer.

Unlike the approaches that recognize only the mouth shape to implement a lip-reading system as [65], there are also models such as Lip2Audspec [91] and Vid2speech [65] that extract speech characteristics from the entire face, not from the mouth area, to synthesize better. Lip2Audspec is a model for reconstructing speech that can be understood in silent lip movement videos using CNN and LSTM, consisting of a lip-reading network and an auto-encoder network. Vid2speech is also an end-to-end model based on CNN to generate understandable speech signals in a speaker’s silent video. All three presented models are deep learning architectures that have made significant progress in the lip-reading field, indicating that AI lip readers can be used to analyze not only in simple speech readings but also users’ real-time thinking [65] and information security areas [96]. While the previously mentioned traditional methods were not universal, deep learning model including LipNet, Lip2Audspec and Vid2speech enabled deeper feature extraction through a general process of extracting lip parts for the image or each frame of the video and processing data through the neural network [94].

### 3.2. Silent Speech Interface

The silent speech interface (SSI) is a system that can produce natural speech and conduct communication through the movement of the tongue, lip, and muscles when auditory signals are still unavailable. The methods of SSI design, which collects and uses these articulatory data to make voice, exist the method of registration-and-synthesis and direct automation-to-speech (ATS) synthesis [97]. When a sensor captures the biosignals from tongue, lip, and muscles, it is analyzed and processed by a digital speech signal, which has been studied to improve speech-handicapped. The recent proliferation of interest in SSI technology began in a very different field, providing privacy for cellular phone conversations because using non-acoustic sensors such as EMA and EMG enabled speech processing even in noisy environments [98]. Therefore, developing an effective algorithm to convert articulatory into speech is the main goal in SSI research [97], and deep learning technology has been introduced to achieve this goal. As the area of speech technology such as speech recognition and speech synthesis using deep learning has become wider, recent studies are attempting to solve the issue of articulatory-to-acoustic conversion [76]. In implementing SSI or silent speech recognition (SSR) technologies, such as sensor handling, interference, and feature extraction, using deep learning are also increasing to improve recognition performance [7]. Recently, DNN has been conducted more frequently than traditional systems, such as Gaussian mixture model (GMM) in speech recognition research, and CNN is also widely used because it proved to be effective in recognizing patterns in the speech signal and image processing [7]. Examples of implementing SSI using deep learning include AlterEgo [99] developed by the MIT Media Lab and SottoVoce [100] developed by the University of Tokyo.

#### 3.2.1. Articulation-to-Speech Synthesis

Articulation is the ability to physically move the tongue, lips, teeth, and chin to produce a series of voice sounds that make up words and sentences. So an articulation-to-speech(ATS) synthesis is underway to restore the original voices of patients who produce abnormal languages, such as speech disorders [101,102,103,104]. Because this technique maps articulatory information directly to speech [104], it is possible to generate voice from articulatory movement data without subject making any sound [103]. In addition to the sensors(EMA, PMA, sEMG) introduced in Section 2, the movement of articulators can be captured using various information, such as ultrasound tongue imaging (UTI), non-audible murmur (NAM), and the advantage of being able to collect voice information, especially from people who can’t make sounds, enables compensation for insufficient training data [77]. In addition, speech synthesis is used as an auxiliary technology for voice communication [77] as a process of inputting text and generating voice waveforms, with the introduction of deep learning in the speech technology area, it is possible to generate more natural voice [76]. So, using a large amount of motion data collected, people who have voice disorders can also synthesize with a natural voice. [102,103,104].

#### 3.2.2. Sensors-AI Integration

**Figure 9:**
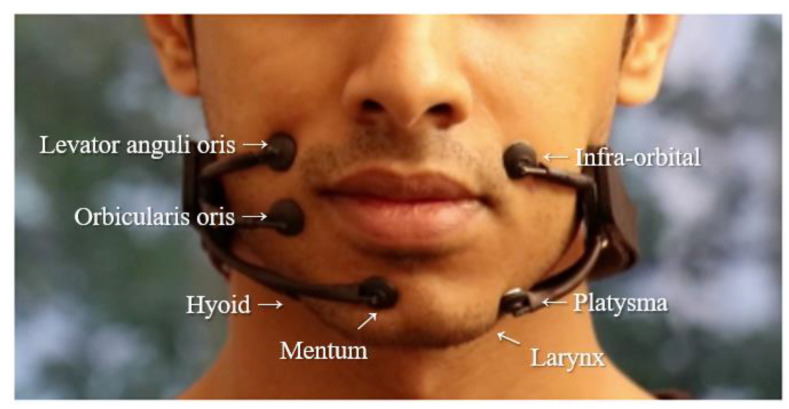
The view of wearing AlterEgo [99].

AlterEgo [99], as depicted in Figure 9, is a wearable silent speech interface that allows bi-directional communication with a computing device without any users’ explicit muscle movement or voice input. AlterEgo does not require any facial and neck muscles to be moved by users but allows AI to find words that respond to signals and communicates among users when they say a certain word in their mouths whisperingly. A non-invasive method was used to capture the users’ neuromuscular signals from the surface of the facial and neck skin via surface EMG that could be attached to seven areas of the skin, including the laryngeal region, hyoid region, and photovoltaic regions. This signal input is an internal vocalization, which described as a characteristic inner voice that can be triggered voluntarily while speaking to oneself [105]. Therefore, the silent speech signals can be obtained without involving brainwave based electrical noise from the frontal lobe. Before being entered into the SSR model, this signal undergoes a representation transformation based on Mel-frequency cepstral coefficient (MFCC), and then the processed signal is classified as a word label, such as numerical digit (0–9) through CNN. When the internal expression is recognized and processed, the external computing device, wirelessly connected over Bluetooth, contextually handles the wording according to the application that the user wants to access. The output calculated is converted to Text-to-Speech (TTS) and then sent to the user via bone conduction headphones. There are limitations that require voluntary input from users, as the platform is not accessible to personal characteristics, although it is strong in terms of extended vocabulary size compared to traditional BCI.

**Figure 10:**
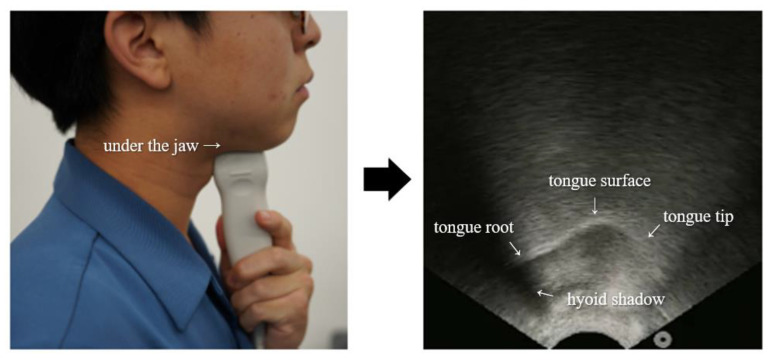
SottoVoce based on ultrasonic image [100].

SottoVoce [100], as depicted in Figure 10, is a silent-speech interaction system that can read the internal oral situation while the user speaks through an ultrasound probe attached to the chin without making a voice. The goal is to reproduce the sound more accurately by transferring ultrasound images into the speech without input voice, so it uses CNN in order to recognize the movement of the tongue that cannot be observed externally. SottoVoce used two neural networks: the first neural network based on CNN uses a series of k ultrasonic images and generates n-dimensional sound-representation vectors and the second neural network generates a sequence of sound-representation vector with the same length for improved sound quality. Since the two networks are speaker-dependent, training requires a series of ultrasound images captured while the user speaks various commands. A series of Mel-scale-spectrum sound-representation vectors, the result of neural network processing, are converted into audio signals through Griffin Lim algorithms and the signals can be used to voice controllable devices such as a smart speaker.

As depicted in Figure 3, digital voicing of silent speech [25] is conducted to turn silently mouthed words into audible speech using EMG. It trains only EMG inputs without time-aligned speech targets, as it collects speech based on EMG sensors generated by the silently articulated speech rather than the vocalized speech. They released a dataset of EMG signals collected during both silent and vocalized speech to find a set of utterances alignments between silent and vocalized speaking modes and associate speech features from silent EMG data. In order to convert EMG input signals into audio outputs, LSTM consisting of three bidirectional LSTM layers with 1024 hidden units is used, and the predicted speech features using alignment are then generated audio using a WaveNet [106] decoder. As a result of the experiment, closed-vocabulary data condition, such as a set of date and time expressions for reading, achieved WER of 3.6%, which is relatively accurate than the 88.8% recorded by direct transfer baseline. In addition, in open vocabulary sentences from books, WER is 68%, which was much lower than in closed vocabulary condition, but still higher than the transfer baseline. When evaluating model performance by comparing 10 electrodes, it was found that location of mid-jaw right, right cheek 2 cm from the nose, back of right cheek, and 4 cm in front of the ear had less effect on model performance.

#### 3.2.3. Deep Learning in Silent Speech Recognition

As shown above, SSI is being studied using a variety of techniques and methods by training many speech data through machine learning and deep learning. SSR using sensors is briefly shown in Table 3. Notable is the fact that current SSR researches have mostly relied on the speaker-dependent recognition models and the number of speakers that can be recognized and classified has become greater than before [107,108,109]. Deep learning-based methods have the advantage of high recognition rates because only specific speakers registered as users can be recognized, but it has the disadvantage of having to go through a training process to a certain extent to prevent overfitting. Thus, it is necessary to develop a speaker-independent SSR [108,110] that can recognize multiple speakers. However, this method also has problems that may vary in recognition performance depending on the speaker, and because it targets unspecified speakers, a large amount of training data will be obligatory. There is also a speaker-adaptation method to compromise between the speaker-dependent and the speaker-independent method, but it has rarely been used in SSR [97]. SSR is also less accurate compared to acoustic speech recognition (ASR) and requires more work [7]. There are two types of issues with recognizing speakers by acoustic speech [108]: verification is the determination of whether the voice sample is the correct voice of a registered speaker’s voice, and identification is the process of finding speaker among several registered candidates when a voice sample is given. One of the biggest difficulties, especially in speech identification, is the quality of the file [111], so as the SSR, and thus research needs to be carried out to classify silent signals with the voice satisfying the voice quality.

## 4. Challenges and Discussion

There are a growing number of studies that use deep learning to recognize speech. Recently, many people use artificial intelligence speakers that can recognize voices. The accuracy of speech recognition techniques is high in restricted environments, but the recognition rate is significantly lower in general environments. An important problem with speech recognition is that it does not work well in noisy environments. AI speakers do not know what commands they will respond to if they have multiple users. And there is also the question of whose authority to respond.

In the case of lip reading using deep learning, it is not easy to accurately measure changes in lip movement. It is difficult to extract features of lips due to external factors such as lighting, skin color, and beard. And it’s hard to tell visually because there are phonemes with the same mouth shape. Also, if the speaker’s posture changes, the angle of the lips changes, which causes problems with recognition accuracy. The problem with the lip-reading method is that it is difficult to carry around the camera for lip recognition.

There is a limit to the fact that speech captured using sensors can only recognize words in the limited vocabulary. Although the ideal auditory feedback latency for a voice is 50 ms [114,115], there is still an unavoidable delay after the speech recognition process to convert input signals to text or output corresponding responses. When the speech recognition system is implemented using sensors, it is not restricted by distance than other biometric technologies such as fingerprints, faces, iris, retina, and veins (based on mobile devices, the recognizable distance is less than 0.3 mm for fingerprints, 8–20 inches for faces, and 25–35 cm for iris scans), but the sensors that can be used to provide intelligent personal agent services are limited, considering portability and feasibility.

This paper shows the possibility of developing speech recognition using sensors in conjunction with deep learning techniques. There are several advantages to speech recognition methods using sensors over other speech recognition methods: (1) Voice recognition can be well done even in noisy environments. (2) Speech recognition is possible without actual sound (silent speech recognition) (3) It is possible to develop a portable product by inserting a voice recognition sensor into a wearable device. (4) Although it is difficult to distinguish user recognition only by difference in voice, it can be improved by utilizing multiple sensors.

**Figure 11:**
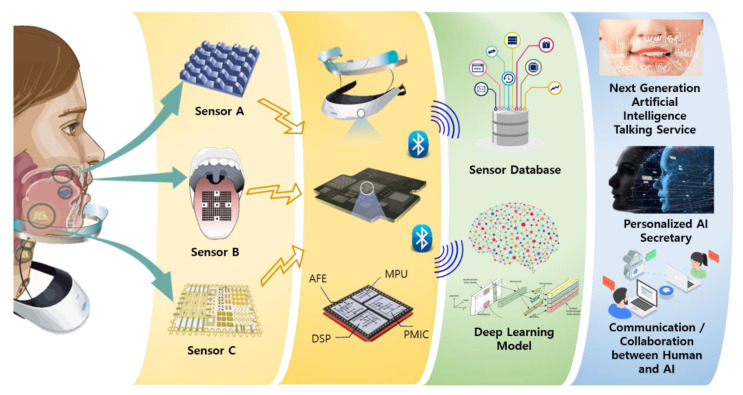
Speech recognition technologies and services using sensor-based deep learning models.

As shown in Figure 11, efficient sensors, such as, are developed and used for speech recognition, and if applied to wearable devices, they can also be applied to various services via deep learning models. Microelectromechanical systems (MEMS) technology is expected to improve performance and mobility, as it can be improved by implementing accelerometers, gyroscopes, pressure sensors, actuators, and signal processing ICs in one-micron size. It can be used by people with voice disorders. However, when speech signals can be obtained at the nerve level using non-invasive BCI devices such as imagined speech or inner speech [9], it is much more difficult to infer the meaning than to recognize speech through muscle movement. Improving speech recognition rates by enhancing a number of these possible problems is a future research challenge. 

## 5. Conclusions

In this review, we have investigated the sensor technologies and the integration of machine learning and deep learning techniques in the field of spoken communication for voice recognition and production. We first introduced methods and techniques to acquire biosignals from muscle activity, brain activity, and articulatory activity as well as their applications in voice recognition and production. It is important for voice recognition technologies to be of high quality and to enable people to express themselves more accurately. In order to overcome the limitations of voice recognition, there have been invented various mouth interface technologies for voice recognition and production with various traditional sensors like EMG, EOG, EEG, EPG, gyro, image, and ultrasonic. 

Recently, deep learning technologies have been introduced to incorporate traditional communication systems and a more advanced voice recognition mechanism. It is also developing into the practical use of voices in various environments and analyzing thoughts in mind in real-time, such as removing noise from data, deciphering various speech-specific pronunciations, and identifying long sentences with context awareness. However, we note that there is a limit to transmitting speech through sound or recognizing speech in the shape of lips. 

We highlighted the issues regarding the tongue positions, lips movements, and voice data with the development devices for the wireless tongue tracking technique combining the several acceleration sensors such as the gyro sensor, the 3-axial magnetic sensor, incorporated with the camera to identify the user’s phonemic markers. Future research requires progress on precise sensors for speech recognition, and the development of deep learning models that enable efficient speech recognition from sensor data is necessary. When a sensor-based voice recognition deep learning model is developed and applied to systems or wearable devices, personalized speech recognition is possible. In the future, artificial intelligence services that can collaborate with humans are available.

## Figures and Tables

**Table 1:** Summary of Speech Recognition Techniques Using Sensors (* Multimodal Speech Capture System).

Application	Organs							References
	Oral Cavity				Muscle		Brain	
	Tongue	Palate	Lip	Larynx	Face	Neck		
**EMG**					√			[15,16,17,18]
					√	√		[6,9,10,15,19,20,21,22,23,24,25]
				√				[26]
EEG							√	[6,8,9,27,28]
EGG				√				[6,9]
EPG	√	√						[9,23,29,30,31,32]
TDS	√	√						[33,34]
MSCS *	√		√					[35]

**Table 2:** Deep Learning Based Speech Recognition Model (* Generative Adversarial Network, ** Convolutional Neural Network based on Transfer Learning, *** Language Model).

Name	Model	Dataset	Result	*Ref.*
Very large DNN models	DNN	2100 h training corpus combining Switchboard and Fisher [78]	- As a result of ASR system performance using up to 400M parameters and 7 hidden layers, structures using NAG [79] optimizer and 3 to 5 hidden layers performed best.	[57]
Robust DNN-HMM	DNN	- Euronews database [79] - APASCI [80]	- The approach based on an asymmetric context window, close-talk supervision, and a supervised close-talk pre-training showed more than 15% performance improvement over the baseline system for contaminated voice training.	[58]
Encoder-Decoder-Attention model	LSTM	- Switchboard 300 h - LibriSpeech 1000 h [81]	Comparing WER, - Switchboard achieved competitive results with existing end-to-end models. - Librispech achieved the WER of 3.54% on the dev-clean and 3.82% on the test-clean subsets, showing the best performance.	[59]
Look-ahead LM ***	RNN	- Wall Street Journal(WSJ) [82,83] - LibriSpeech [81]	- The compared result with other end-to- end systems, 5.1% WER for WSJ eval92 and 8.4% WER for WSJ dev93. - When comparing WER with other language models, the model obtained consistent error reduction as the size of the vocabulary increased.	[60]
LSTM RNN acoustic models	LSTM RNN	3 million utterances with an average duration of about 4 s, taken from real 16 kHz Google voice search traffic	- Models using the state-level minimum Bayes risk sequence discriminative training criterion [84] have achieved continuous WER improvement.	[61]
AENet	CNN	- Freesound [85] to create a novel audio event classification database - USF101 dataset [86] to evaluate the AENet features	- Recognizing the variety of audio from theevent, the audio event detection capability has improved by 16% and video highlight detection by more than 8% compared to the commonly used audio features.	[62]
Deep Speech2	RNN CNN	- English: 11,940 h of speech 8 million utterances - Mandarin: 9400 h of speech 11 million utterances	- English: 2 layers of 2D CNN, 3 layers of unidirectional RNN. - Mandarin: 9 layers of 7 RNN with 2D convolution and BatchNorm.	[63]
LipNet	CNN GRU	GRID corpus [87]	- The accuracy of the sense-level in the GRID dataset is 95.2%.	[64]
Vid2speech	CNN	GRID corpus	- The audio-visual test using Amazon MTurk having word intelligibility of 79%.	[65]
AIPNet	GAN * LSTM	9 English accents containing 4M (3.8K h) utterances crowd-sourced workers	- Supervised setting achieved 2.3~4.5% relative reduction on WER with *L_ASR_*. - Semi-supervised setting achieving 3.4~11.3% WER reduction.	[66]
Parrotron	LSTM CNN	30,000 h training set 24 million English utterances	WER of 32.7% from a deaf speaker with nonsense words	[67]
TLCNN **-RBM	CNN RBM	NIST 2008 SRE dataset [88], self-built speech database, TIMIT dataset [89]	- CNN, which has FBN, reduces training time by 48.04% compared to CNN without FBN. - 97% higher average accuracy than when using CNN or the TL-CNN network.	[68]
Lip reading model	CNN LSTM	audio-visual database with 10 independent digital English utterances	- The accuracy is 88.2% in the test dataset.	[90]
Lip2Audspec	CNN LSTM	GRID corpus [87]	- The average accuracy of 20 workers is 55.8%.	[91]

**Table 3:** Deep Learning Based Silent Speech Recognition (* Silent Speech Challenges, ** Deep Canonical Correlation Analysis, *** Feature space Maximum Likelihood Linear Regression).

Model	Method	Data	Result	*Ref.*
DNN-HMM to reduce the WER compared to the GMM-HMM approach used in SSC *	Ultrasonic probe (tongue), Infrared-illuminated video camera (lip)	- SSC data recorded without any vocalization - 320 × 240 pixel tongue images and 640 × 480 pixel lip images in black and white	WER of 6.4% is obtained, which is lower than the published benchmark [112] value of 17.4%.	[7]
Voicing Silent Speech	EMG	20 h of facial EMG signals from a single speaker collected during both silent and vocalized speech	WER of 3.6% from closed-vocabulary data condition and 68% from the open vocabulary condition.	[25]
AlterEgo	attaching electrodes to neuromuscular muscles	Synthetic data corpus	The average word accuracy of 10 users is 92.01%.	[99]
SottoVoce	Ultrasonic probe	- rescaled ultrasonic image with 500 speech commands	- The success rate of recognizing the smart speaker (Amazon Echo) is 65%.	[100]
DCCA ** to find the correlation between articulatory movement data and acoustic features	Electromagnetic Articulograph	- speaker-independent (7 speakers) - 3-dimensional movement data of articulators (tongue and lip) - included acoustic data	the Phoneme error rate of 57.3% using only DNN-HMM, which is 45.9% when combined with DCCA and 42.5% when combined with DCCA + fMLLR ***.	[113]
